# Contributing to the management of viral infections through simple immunosensing of the arachidonic acid serum level

**DOI:** 10.1007/s00604-024-06440-y

**Published:** 2024-06-04

**Authors:** Rebeca M. Torrente-Rodríguez, Víctor Ruiz-Valdepeñas Montiel, Simona Iftimie, Ana Montero-Calle, José M. Pingarrón, Antoni Castro, Jordi Camps, Rodrigo Barderas, Susana Campuzano, Jorge Joven

**Affiliations:** 1 Departamento de Química Analítica, Facultad de CC. Químicas, Universidad Complutense de Madrid, Pza. de las Ciencias 2, Madrid, 28040 Spain; 2 Servei de Medicina Interna, Hospital Universitari de Sant Joan, Institut d’Investigació Sanitària Pere Virgili, Universitat Rovira i Virgili, Av. Dr. Josep Laporte 2, Reus, 43204 Spain; 3 Chronic Disease Programme, UFIEC, Instituto de Salud Carlos III, Majadahonda, Madrid, 28220 Spain; 4 Unitat de Recerca Biomèdica, Hospital Universitari de Sant Joan, Institut d’Investigació Sanitària Pere Virgili, Universitat Rovira i Virgili, Av. Dr. Josep Laporte 2, Reus, 43204 Spain; 5 CIBER of Frailty and Healthy Aging (CIBERFES), Madrid, Spain

**Keywords:** Electrochemical bioplatform, Amperometry, Screen-printed carbon electrode, Arachidonic acid, Serum samples, SARS-CoV-2, RSV

## Abstract

A trendsetting direct competitive-based biosensing tool has been developed and implemented for the determination of the polyunsaturated fatty acid arachidonic acid (ARA), a highly significant biological regulator with decisive roles in viral infections. The designed methodology involves a competitive reaction between the target endogenous ARA and a biotin-ARA competitor for the recognition sites of anti-ARA antibodies covalently attached to the surface of carboxylic acid-coated magnetic microbeads (HOOC-MµBs), followed by the enzymatic label of the biotin-ARA residues with streptavidin-horseradish peroxidase (Strep-HRP) conjugate. The resulting bioconjugates were magnetically trapped onto the sensing surface of disposable screen-printed carbon transducers (SPCEs) to monitor the extent of the biorecognition reaction through amperometry. The operational functioning of the exhaustively optimized and characterized immunosensing bioplatform was highly convenient for the quantitative determination of ARA in serum samples from severe acute respiratory syndrome coronavirus 2 (SARS-CoV-2-) and respiratory syncytial virus (RSV)-infected individuals in a rapid, affordable, trustful, and sensitive manner.

**Graphical Abstract:**

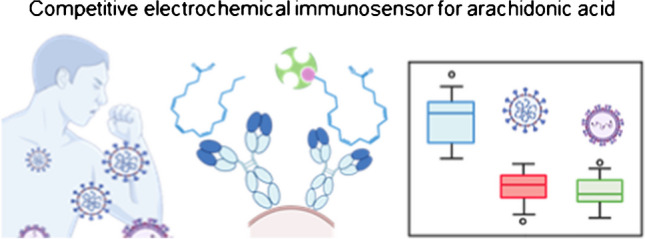

**Supplementary information:**

The online version contains supplementary material available at 10.1007/s00604-024-06440-y.

## Introduction

Arachidonic acid (ARA or AA, 20:4, n-6) is a ω-6 polyunsaturated fatty acid with a fundamental role in human health and several diseases [1–3]. There is a high content of free ARA in the human body, which generally comes from animal sources (meat, eggs, and dairy) or is converted from linoleic acid [1].

ARA plays an important role in the regulation of a wide variety of important physiological processes such as preventing cardiovascular diseases, diabetes, and tumors, reducing blood viscosity, and improving intelligence and memory [4]. On the other hand, numerous investigations correlate the dysregulation in ARA levels with inflammatory processes, heart diseases [5, 6], high blood pressure, acute intracerebral hemorrhage [7], Alzheimer’s disease, lupus, arthritis [4], HIV [8], COVID-19 [9–14], and prostate cancer [15].

It has been reported that ARA, other unsaturated fatty acids, and some of their metabolites can serve as endogenous antimicrobial compounds and their deficiency can make humans susceptible to infections with SARS-CoV-2, SARS, MERS, and other similar enveloped viruses [11, 16–20]. Recent research also shows that ARA activates human voltage-gated proton channels in infected, injured, or inflamed cells [21]. Therefore, it is currently considered that the oral or intravenous administration of these fatty acids can improve recovery from these viral infections and that their presence in adequate quantities in the immunocytes and body fluids (especially in the alveolar fluid) can prevent these infections or at least reduce their severity [18, 22].

All of the above highlights the need to have analytical tools capable of determining ARA levels in humans. Reported methods for the determination of ARA include liquid chromatography coupled with mass spectrometry (LC-MS and LC-MS/MS), high-performance liquid chromatography coupled with fluorescence detection, near-infrared spectroscopy, and enzyme-linked immunosorbent assay (ELISA) [1, 4]. These methods provide good sensitivity and precision, but their operation is complicated and requires a long time, delicate instruments, and sample pretreatment. However, it is surprising that electrochemical sensors and biosensors, which are characterized by their simplicity, fast response times, and feasibility for incorporation into robust, portable, low-cost, and miniaturized devices, have hardly been used for the determination of ARA. To our knowledge, only one electrochemical sensor has been reported for the voltammetric determination of ARA using a glassy carbon electrode (GCE) modified with a small polypeptide of arginine-glycine-aspartic doped with AuNPs (RGD-Au). The RGD-Au-GCE measured the electrochemical signal obtained from the reduction of 1,4-naphthoquinone in the presence of ARA as a proton source. A detection limit of 80 nM ARA was reported, and satisfactory results were found in the analysis of serum samples from healthy individuals [4].

Recent studies from Castañe et al. [11] reported that serum concentrations of ARA measured by targeted lipidomics (ultra-high-pressure liquid chromatography coupled to a quadrupole-time-of-flight mass spectrometer and a dual jet stream electrospray ionization) served as a robust marker of SARS-CoV-2 infection. This work prompted our investigation into the development of a rapid and straightforward method for determining serum ARA levels. This pursuit is motivated by the current inadequacies in analytical options for diagnosing, prognosticating, and monitoring viral infections. The timely identification of severe cases, individuals, and regions susceptible to infectious diseases is crucial for prompt health interventions and effective surveillance. In this context, the identification of specific parameters that allow a more precise diagnosis and evaluation of the severity of viral infections, and the development of new technologies that allow their rapid, simple, reliable, affordable, and in situ determination, is considered imperative. These actions will carry significant benefits in mitigating the risk of inadequate antimicrobial treatments, optimizing clinical outcomes, minimizing toxicity and other adverse events, and reducing healthcare expenditures associated with infections and mitigating the emergence of antimicrobial-resistant strains [23].

Considering the capabilities that electrochemical affinity bioplatforms have demonstrated [24] and particularly those combining the advantages of using magnetic microparticles (MµBs) [25] and amperometric transduction on screen-printed electrodes (SPCEs), we report in this work the first immunoplatform for the determination of ARA. The developed biotool involves a direct competitive immunoassay between ARA and biotinylated ARA for the limited binding sites of immunoconjugates prepared by covalent immobilization of a selective antibody on MµBs functionalized with carboxylic groups through succinimide/hydroxysuccinimide chemistry. After enzymatic labeling of the biotinylated ARA captured on the MµBs using a commercial streptavidin-horseradish peroxidase (Strep-HRP) conjugate, amperometric transduction was performed in the presence of the hydroquinone (Hq)/H_2_O_2_ system by trapping the resulting magnetic bioconjugates on the working electrode of a SPCE. The variation in the measured current was inversely dependent on the ARA concentration in the sample. The developed bioplatform was applied to the analysis of serum samples from healthy individuals and from patients positive for SARS-CoV-2 and respiratory syncytial virus (RSV) showing a lower ARA serum level in infected patients.

## Experimental

### Apparatus and electrodes

Signal transduction was carried out by amperometry at room temperature with a CHI812B potentiostat (CH Instruments, Inc.) controlled by the CHI812B software, and using screen-printed carbon electrodes (SPCEs, DRP110) containing a carbon working electrode (WE, 4-mm Ø, working electrode active area 12.6 mm^2^), a carbon auxiliary electrode (AE), and a silver pseudo-reference electrode (RE) and their corresponding cable connector (DRP-CAC), purchased from Metrohm-DropSens. A µAutolab type III potentiostat (Ecochemie) controlled by FRA2 software electrochemical impedance spectroscopy (EIS) was employed for recording electrochemical impedance spectra (EIS) in 5 mM [Fe(CN)_6_]^−3/−4^ in 0.1 M KCl solutions.

Resultant magnetic bioconjugates were reproducibly trapped onto the surface of SPCE with the help of a lab made polymethylmethacrylate (PMMA) casing comprising a 4-mm Ø neodymium (Nd) magnet (AIMAN GZ).

The proper sequential modification of MµBs was performed using a Dynamag-2 Magnet magnetic separator (Invitrogen-ThermoFisher Scientific). Other employed apparatus included a vortex (VELP Scientifica), a Basic 20+ (Crison) pH-meter, and a thermomixer MT100 constant temperature incubator shaker (Universal Labortechnik).

### Reagents and solutions

All used reagents were of the highest analytical grade. Carboxylated and streptavidin coated magnetic microbeads (MµBs) (HOOC-MµBs, 2.8 µm Ø, 2 × 10^9^ beads mL^−1^, Dynabeads™ M-270 carboxylic acid, 14305D and Strep-MµBs, 2.8 µm Ø, 10 mg mL^−1^, Dynabeads M-280 Streptavidin, 11206D, respectively) were acquired from Invitrogen-ThermoFisher™ (Waltham, MA, USA). Different salts, including 2-(N-morpholino)ethanesulfonic acid (MES), Tris-hydroxymethyl-aminomethane-HCl (Tris-HCl), sodium and potassium chloride (NaCl and KCl, respectively), sodium di-hydrogen phosphate (NaH_2_PO_4_), and disodium hydrogen phosphate (Na_2_HPO_4_), were purchased from Scharlab (Barcelona, Spain). Required reagents to perform activation and blockage of HOOC-MµBs as well as to perform the amperometric transduction included N-hydroxysulfosuccinimide (Sulfo-NHS), N(3-dimethyl aminopropyl)-N’-ethyl-carbodiimide (EDC-HCl), ethanolamine (Et), hydrogen peroxide (H_2_Subscript>O_2_, 30% (w/v)), and hydroquinone (Hq) were provided by Sigma-Aldrich (Saint Louis, MS, USA). Commercial Blocker™ Casein in PBS (blocking buffer, BB) was from Thermo Scientific, and streptavidin-horseradish peroxidase conjugate (Strep-HRP, 500 U mL^−1^) was from Sigma-Aldrich.

The immunoreagents supplied by ELISA Kit DIY Materials for Arachidonic Acid (AA) (Product No. KSB098Ge11, Cloud-Clone Corp., Houston, TX, USA) containing specific antibody (anti-ARA Ab), standard (ARA), and biotin-labeled ARA (biotin-ARA competitor) were employed for constructing the developed immunoplatform. Biotin-labeled ARA antibody (from ELISA kit Ref. NBP2-66372) and ARA standard and HRP-labeled ARA (from ELISA kit Ref. NBP2-59872) both acquired from NOVUS were also evaluated as immunoreagents.

Immunoglobulin G from human serum (hIgG, Ref: I2511), human serum albumin (HSA, Cat. No. A1653), and cholesterol (Cho, Ref: C8667) were tested as non-targeted compounds and were purchased from Sigma-Aldrich.

Buffered solutions of phosphate-buffer saline (PBS) containing 137 mM NaCl and 2.7 mM KCl (pH 7.5), 0.025 M MES (pH 5.0), 0.1 M phosphate buffer solution (PB) (pH 8.0), 0.1 M Tris-HCl (pH 7.2), and 0.05 M sodium phosphate buffer (PB) (pH 6.0) were prepared using type I deionized water from a Millipore (Burlington, MA, USA) water purified by Milli-Q purification system (18.2 MΩ cm). Activation and blocking step of HOOC-MµBs required EDC-HCl/Sulfo-NHS mixture (50 mg mL^−1^ each) and Et (1.0 M) solutions prepared in MES (pH 5.0) and PB (pH 8.0), respectively. 0.1 M stock solutions of the redox probe (Hq) and of the HRP enzyme substrate (H_2_O_2_), required to perform the amperometric detection, were freshly prepared in 0.05 M PB (pH 6.0).

### Assemblage of the immunostrategy and amperometry-based detection

The biofunctionalization of HOOC-MµBs consisted of sequential 25 µL-incubation steps at 25 °C and 950 rpm in 1.5-mL microcentrifuge tubes. When each incubation step concluded, 50 µL-washing steps were performed before the next incubation step by placing the microcentrifuge tube in a magnetic concentrator for 3 min and discarding the supernatant solution.

The preparation of the bioplatform started by pipetting 1 µL of commercially available HOOC-MµBs suspension into a microcentrifuge tube and followed by two washings with MES buffer for 10 min each. Next, derivatization of the carboxylic acid residues coating the MµBs was carried out by their re-suspension in EDC-HCl/Sulfo-NHS mixture solution for 35 min. After two washings with MES buffer, anti-ARA Ab molecules were covalently bounded to the derivatized MµBs by incubation in a 25-µg mL^−1^ anti-ARA Ab solution for 45 min, followed by two washings with MES buffer. A 60 min blocking incubation step with Et solution was performed to avoid unspecific adsorptions. The blocked MµBs were washed with Tris-HCl buffer and twice with BB. The anti-ARA Ab-MµBs were stored in filtered PBS buffer solution at 4 °C.

Direct competition between free ARA and biotin-ARA competitor for the available binding sites of anti-ARA Ab molecules was carried out by incubating for 45 min anti-ARA Ab-MµBs in a mixture solution in BB containing the desired concentration of ARA standard (or the analyzed sample) and 0.1 µg mL^−1^ biotin-ARA competitor. After two washings with BB, enzymatic label of the captured biotin-ARA competitor onto the surface of the MµBs was performed by incubating for 15 min the magnetic conjugates in a 1/1000 Strep-HRP solution prepared in BB, followed by two washings with the same buffer.

Subsequently, the resulting magnetic conjugates were suspended in a 50 µL PB buffer (pH 6.0) aliquot and pipetted onto the surface of the WE of a SPCE previously fitted into the lab-made Nd magnet-PMMA casing. To carry out the amperometric measurements, the ensemble SPCE/magnetic casing was connected to the potentiostat through the corresponding cable connector and immersed into an electrochemical cell containing 10 mL of 0.05 M PB (pH 6.0) and 1.0 mM of Hq under magnetic stirring. Next, a constant potential of − 0.20 V (vs. Ag pseudo-reference electrode) was applied to the WE, and when the background current was stabilized, a 50 µL aliquot of the freshly prepared H_2_O_2_ solution was added to the electrochemical cell. The resultant cathodic current was measured until reaching the steady state, and the corresponding signal magnitude was calculated as the difference between the steady state and background currents. Unless otherwise specified, the mean values of 3 replicates and error bars estimated as three times the standard deviation (SD) are given.

### Analysis of serum samples from healthy and RSV and SARS-CoV-2-infected patients

Serum samples from healthy, RSV- and SARS-CoV-2-infected individuals (*n* = 10 for each group) were provided by the Hospital Universitari Sant Joan de Reus (Tarragona) after approval of the corresponding Ethical Committee (Resolution 040/2018 amended on 16 April 2020). All individuals and patients involved in the study voluntarily signed and consented the use of their serum samples for research purposes. All patients were treated in the hospitalization ward, and samples were collected in 2021. As established guidelines for safe handling, all virus-containing human serum samples were previously inactivated at high temperature (55 °C for 30 min) to guarantee safe and zero risk handling by the operator. After the inactivation treatment, serum samples were stored at − 20 °C until analysis.

The existence of matrix effect was evaluated by comparing the slope values from the linear least-squared regression obtained with the developed bioplatforms in buffered solution and in a 10-times diluted representative serum sample from a SARS-CoV-2-infected patient. Once demonstrated that matrix effect was negligible for 10-times diluted serum samples, quantification of endogenous free ARA was performed through simple interpolation of the cathodic current variation measured with the developed amperometric bioplatform into the calibration curve constructed with standards. The same samples were analyzed with the commercial ELISA kit (Product No. KSB098Ge11, Cloud-Clone Corp., Houston, TX, USA).

### Statistical analysis

Mann-Whitney non-parametric test values were calculated with R (version 3.6.2). *P*-values ≤ 0.05 were considered statistically significant. Receiver Operating Characteristic (ROC) curves were constructed with R and the areas under the curve (AUC), specificity, sensitivity, and cut-off values calculated with R, using the “ModelGood” and the “Epi” packages.

## Results and discussion

**Fig. 1:**
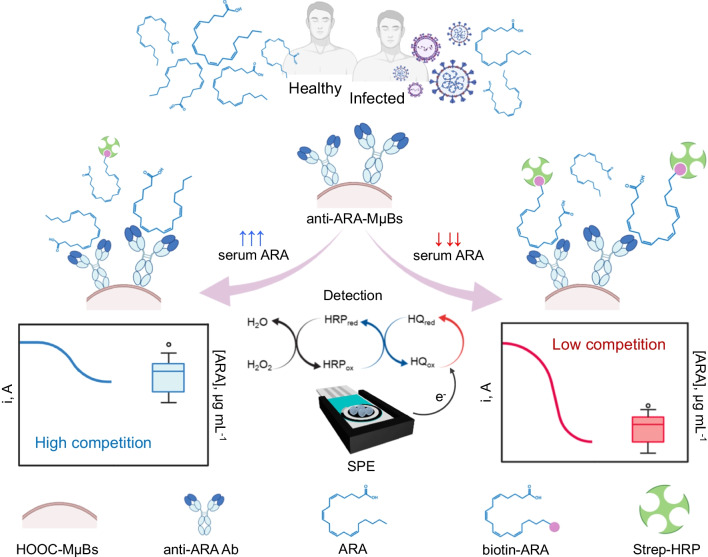
Schematic diagram of the method developed for the determination of ARA

The rational combination of all different biocomponents to implement the direct-competitive immunosensing strategy for the quantification of ARA is depicted in Fig. 1. The biosensing method relied on the competition reaction between endogenous ARA and the chemically functionalized ARA (biotin-ARA competitor) for the available binding sites of specific anti-ARA immunoglobulin G antibodies (anti-ARA Ab) covalently linked onto the surface of magnetic micro-sized beads coated with carboxylic functional groups (HOOC-MµBs). Thereafter, the enzymatic labeling of the captured biotin-ARA residues with streptavidin-horseradish peroxidase (Strep-HRP) conjugate was carried out. The resultant bioconjugates were magnetically trapped on the surface of the working disposable SPCE previously coupled with a lab designed PMMA casing containing a neodymium magnet placed under the working sensing surface. HRP/H_2_O_2_/hydroquinone (Hq)-based amperometry at − 0.20 V (vs. Ag pseudo-reference electrode) in stirred solutions was carried out to monitor the extent of the affinity reactions. According to the fundamentals of direct competitive-based immunoassays, the recorded amperometric responses resulted inversely proportional to the concentration of the free ARA biomarker.

### Influence of the selected immunoreagents on assay viability

A careful selection of the proper immunoreagents was accomplished. In fact, not only the selectivity but also the sensitivity and dynamic working range of developed biosensor are dependent on the chosen immunoreagents. The adequate selection of immunoreagents is so important that it is not advisable to compare results obtained by different techniques using different reagents, since the recognition abilities and antigen heterogeneity may be responsible for inter-methods differences [26–28]. Therefore, amperometric measurements obtained with direct-competitive immunostrategies involving different available capture antibodies, labeled probes, and solid supports, in the absence and in the presence of a fixed concentration of ARA, were compared. Different protocols were tested: (I) covalent immobilization of anti-ARA Ab onto previously activated and blocked HOOC MµBs followed by sequential incubation steps in a solution containing biotin-ARA competitor and a Strep-HRP conjugate; (II) immobilization of biotin-anti-ARA Ab onto the surface of Strep-MµBs followed by an incubation step with HRP-labeled ARA competitor; and (III) covalent immobilization of anti-ARA Ab molecules onto previously activated and blocked HOOC-MµBs followed by an incubation step in a solution containing HRP-labeled ARA competitor.

**Fig. 2:**
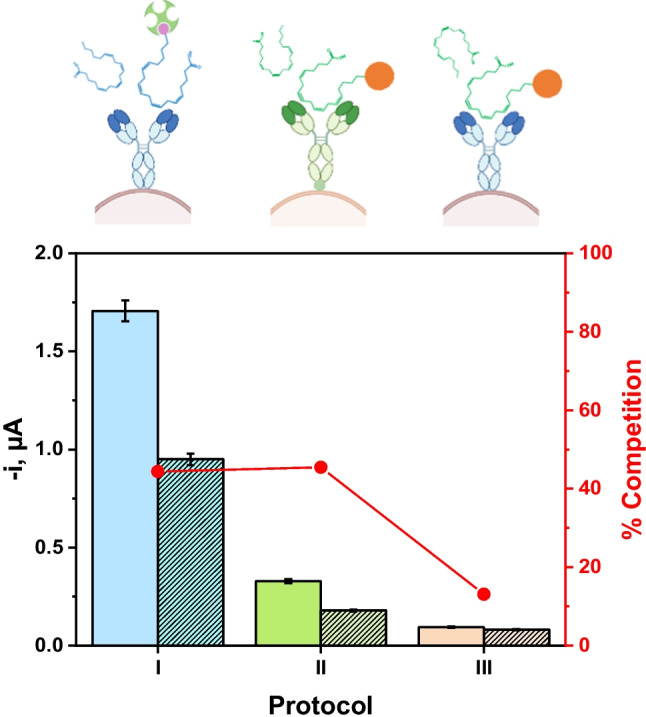
Influence of the selection of immunoreagents on the performance of the direct competitive biosensor for determination of ARA. Dependence of the cathodic current with the corresponding competitive assay protocol (illustrated schemes at the top) involving (I) anti-ARA Ab, biotin-ARA competitor, and Strep-HRP enzymatic conjugate, (II) biotin-anti-ARA Ab and HRP-labeled ARA competitor, and (III) combination of anti-ARA Ab and HRP-labeled ARA competitor. HOOC- (I and III) or Strep- (II) MµBs were used as solid supports in the absence (non-patterned bars) and the presence (patterned bars) of 2.5 µg mL^−1^ ARA standards. The corresponding competition percentage is displayed as red dots connected by lines for the bioplatform prepared according to each tested protocol. The experimental conditions for each protocol were 1.0 µL of HOOC-MµBs, 25 µg mL^−1^/45 min incubation time/25 °C for anti-ARA Ab, 0.1 µg mL^−1^ biotin-ARA competitor/45 min incubation time/25 °C, and 1/1000 Strep-HRP enzymatic conjugate/15 min incubation time/25 °C (Protocol I); 5.0 µL of Strep-MµBs, 1/50 biotin-anti-ARA Ab/60 min incubation time/37 °C, and 1/50 HRP-labeled ARA competitor/30 min incubation time/37 °C (Protocol II); 1.0 µL HOOC-MµBs, 25 µg mL^−1^/45 min incubation time/25 °C for anti-ARA Ab, and 1/50 HRP-labeled ARA competitor/45 min incubation time/25 °C (Protocol III)

The results displayed in Fig. 2 show that both the amperometric responses and competition percentages were significantly lower using protocol III. This was attributed to a lack of recognition between the immunoreagents (anti-ARA Ab and HRP-labeled ARA competitor) from different ELISA kits (bar III). Therefore, this design was discarded for developing the biosensing platform. On the other hand, rather similar and acceptable competition percentages ($\sim$44–45%) were obtained when protocols I and II were applied. A clear discrimination between the amperometric currents recorded in the absence and in the presence of 2.5 µg mL^−1^ ARA was observed, and, therefore, comparable final operational performance for the bioplatforms constructed with the reagents supplied with both ELISA kits should be expected. Nevertheless, protocol I was selected for the design of the bioplatform because of the reached sizeable maximum signals, which is known as a prerequisite condition to widen the linear working range in competitive-based immunostrategies.

### Optimization of key experimental variables affecting the bioplatform efficiency

Before assessing the different variables affecting the analytical features of the designed methodology, control experiments were carried out in PBS and BB buffered media. The amperometric responses obtained in the absence (blank, B) and in the presence (target, T) of 5.0 µg mL^−1^ ARA on unmodified and anti-ARA Ab HOOC-MµBs were compared. The results displayed in Fig. S1 (in the Supporting Information) indicated that the amperometric readings obtained in PBS solutions were similar when the assay was accomplished onto unmodified or modified HOOC-MµBs due to the significant non-specific adsorption of biotin-ARA residues onto the surface of the unmodified HOOC-MµBs. The substantial reduction of the non-specific binding of immunoreagents and thus the improvement of blank-to-target ratio (B/T), as well as the enhancement in discrimination between the absence and the presence of ARA, can be solved by using blocking agents, such as BB, as it has been reported for other biosensing architectures [29, 30]. The effectiveness of the BB solution as well as the suitability of the proposed methodology was proven by considering the B/T ratios obtained using unmodified (B/T = 0.97) and modified (B/T = 2.06) HOOC-MµBs. Therefore, the BB solution was chosen as buffer solution for subsequent optimization studies.

Next, the variables affecting the bioplatform preparation were checked and univariately optimized. Considering the immunoassay configuration, larger blank-to-target ratio (B/T) between the amperometric responses in the absence (B) and in the presence (T) of 5.0 µg mL^−1^ ARA standards was taken as the selection criterion. The set of evaluated and selected conditions for the construction of the bioplatform for ARA determination are summarized in Table 1 and depicted in Fig. S2 (in the Supporting Information), respectively.

**Table 1:** Summary of the experimental variables, checked ranges, and selected values for developing competitive bioplatforms for the determination of ARA

Variable	Range studied	Selected value
Buffer	PBS and BB	BB
Volume HOOC-MµBs, µL	1–5	1
[anti-ARA Ab], µg mL^−1^	0.0–50	25
Incubation time _anti−ARA Ab_, min	15–60	45
Analysis steps	1–2	2
[biotin-ARA competitor], µg mL^−1^	0.01–2.5	0.1
Incubation time _ARA & biotin−ARA competition_, min	15–60	45
Strep-HRP, dilution	1/10,000–1/500	1/1000
Incubation time _Strep−HRP_, min	15–60	15

Firstly, the effect of the MµBs suspension volume on the response provided by the developed bioplatforms was evaluated. As Fig. S2a shows, the competition between free ARA and biotin-ARA competitor was possible to be measured for the lowest volume of HOOC-MµBs suspension (1 µL). Larger volumes (2–5 µL) produced a dramatic decrease of the B/T ratio mainly due to the decrease in the amperometric blank responses. This effect can be attributed to the higher electron transfer resistance with large MµBs loadings and/or the increase of the diffusion barrier on the WE surface [31], as confirmed the experimental results displayed in Fig. S3 (in the Supporting Information). On the other hand, according to Fig. S2b, 5.0 µg mL^−1^ anti-ARA Ab is not enough to discriminate the presence of ARA due to the limited amount of immobilized antibody [32]. However, the B/T ratio reached a larger value for 25 µg mL^−1^ and dramatically decreased for higher antibody loadings, probably due to a worse recognition of biotin-ARA because of steric hindrance. Therefore, 1 µL MµBs suspension and 25 µg mL^−1^ anti-ARA Ab were chosen for further work. It is important to highlight that the lowest measured currents were obtained for 0.0 and 5.0 µg mL^−1^ ARA when no anti-ARA Ab was attached to the MµBs (bars 0.0 in Fig. S2b), thus confirming the absence of non-specific adsorptions for ARA, the competitor biotin-ARA, and the enzymatic tracer Strep-HRP, as well as the suitability of the assay. The incubation time for anti-ARA Ab immobilization was evaluated over the 15 to 60 min range (Fig. S2c); 45 min was selected because this incubation period provided a larger B/T ratio.

Another parameter of paramount relevance in the practical performance of biosensing tools is the number of steps needed for implementing the methodology. In this case, two different assay protocols (both starting from the blocked anti-ARA Ab-MµBs and involving 30-min incubation steps with the corresponding solutions) were evaluated (Fig. S2d):

i) One-step protocol by incubation with a mixture solution containing biotin-ARA and Strep-HRP in the absence (B) or in the presence (T) of 5.0 µg mL^−1^ of free ARA standard,
ii) Two-step protocol consisting of incubation of biotin-ARA competitors in the absence (B) or in the presence (T) of 5.0 µg mL^−1^ of free ARA standard, followed by the enzymatic labeling with Strep-HRP of the attached biotin-ARA conjugates.

Figure S2d shows that the 2-step protocol provided a larger B/T ratio due to the significant decrease in the blank signal when the capture and the enzymatic labeling of the biotin-ARA competitors occurred simultaneously, which is probably due to a less favorable immunorecognition caused by steric hindrance effects.

Concerning the concentration of the biotin-ARA competitor (Fig. S2e), both the amperometric currents obtained in the absence (B) and in the presence (T) of ARA increased with biotin-ARA concentration. This increase was more pronounced for B resulting in a progressive increase of the B/T ratio up to a concentration of 0.5 µg mL^−1^. Above this value, a sharp decrease in B/T values was observed due to a worse competition in the presence of a high concentration of the competitor [32, 33]. Since the discrimination for ARA was not significantly improved from 0.1 to 0.5 µg mL^−1^ biotin-ARA competitor, a 0.1 µg mL^−1^ concentration was selected for further work to economize the methodology. The incubation time of the mixture solution containing the ARA standard concentration and 0.1 µg mL^−1^ (Fig. S2f) showed that a larger B/T ratio was obtained for 45 min. In addition, variables directly affecting the enzymatic tracer such as the dilution factor and incubation time were checked. Fig. S2g shows that a better B/T ratio was found for a Strep-HRP 1/1000 dilution. Although Fig. S2h shows a slightly larger B/T ratio for a 30-min incubation, a 15-min period was selected because no significant decrease of the ratio was apparent for this shorter incubation time. Interestingly, the lowest Strep-HRP dilution and the largest incubation time checked hurt the competition for a fixed ARA concentration most likely because an excess of enzymatic probes impeded the detection of a relatively low concentration of free ARA in solution. Other variables related to the amperometric detection, such as the applied potential and the concentration of both the Hq redox probe and the enzymatic substrate (H_2_O_2_), were optimized in previous works [34, 35].

The optimizations of the variables affecting the performance of the developed bioplatform allowed the determination of this very relevant yet scarcely explored polyunsaturated fatty acid in just 60 min, which is remarkably faster than that claimed using ELISA.

### Analytical and operational features for the determination of ARA

**Fig. 3:**
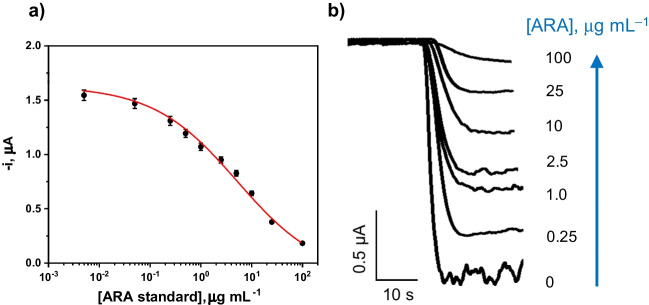
Calibration curve (**a**) and representative real amperometric traces (**b**) obtained with the developed bioplatform for the determination of ARA

The dependence of the amperometric responses obtained with the developed bioplatform for increasing concentrations of ARA standards is plotted in Fig. 3. The results fitted to a sigmoidal curve, as expected for competitive immunoassays, according to the equation:

$$y={i}_{min}+\frac{\left({i}_{max}-{i}_{min}\right)}{1+{\left(\frac{x}{{EC}_{50}}\right)}^{-p}}$$

The parameters of the sigmoidal equation included *y* as the amperometric reading, *x* as the ARA concentration (in µg mL^−1^), *i*_*min*_ as the lowest amperometric signal obtained for a hypothetical infinite concentration of ARA target, *i*_*max*_ as the highest amperometric signal obtained in the absence of ARA target, *EC*_*50*_ as the concentration causing an *i*_*max*_ decrease by half, and *p* as the Hill’s slope of the calibration curve. The values of these parameters as well as the linear range and LOD, calculated according to the ARA concentration giving rise to a 10% decay of the *i*_*max*_, are summarized in Table 2.

**Table 2:** Fit-related parameters corresponding to the calibration curve shown in Fig. 3 obtained with the developed bioplatform

Fit-related parameters	Value
*r*^2^	0.995
EC_50_, µg mL^−1^	5.544
Dynamic range, µg mL^−1^	0.4–78
LOD, µg mL^−1^	0.084
LOQ, µg mL^−1^	0.393

It is important to remark that, according to the reported levels of five polyunsaturated fatty acids (PUFA) in human serum, the working range achieved with the developed immunoplatform is suitable for the analysis of ARA since its mean level in healthy individuals was claimed to be 46.4 µg mL^−1^ [36]. The LOD achieved with the developed method (84 ng mL^−1^ or 263 nM) is 10 times lower than that claimed for the ELISA kit employing the same immunoreagents (0.88 µg mL^−1^; CEB098Ge) and similar to those provided by other commercial ELISA kits (69.7 ng mL^−1^ (EH4021 from Fine Test); 78.1 ng mL^−1^ (LS-F10090 from LSBio)). It is also important to highlight that although the LOD provided by the developed bioplatform is about three times larger than that claimed for the only electrochemical sensor reported to date for the determination of ARA (80 nM [4]), this sensor used conventional electrodes modified with a polypeptide doped with AuNPs (a nanomaterial that requires more than 12 h for preparation) and was only applied to the determination in serum samples from healthy individuals.

The reproducibility of the amperometric measurements provided by the developed immunoplatform for 5.0 µg mL^−1^ ARA standards was tested. A relative standard deviation (RSD) value of 3.1% was obtained from the measurements carried out with 6 different bioplatforms prepared following the same protocol, which allows us to conclude that a good operational reproducibility, including both the bioplatform preparation protocol and the amperometric transduction on the SPCE, was achieved.

In addition, the storage stability of anti-ARA Ab-HOOC-MµBs was checked by preparing and storing a batch in filtered PBS at 4 °C. The comparison of the B/T ratios for 5.0 µg mL^−1^ ARA standards on the day of the preparation of the magnetic bioconjugates (day 0) and on subsequent days (data not showed) demonstrated that there were no significant differences for 5 days.

### Evaluation of the selectivity of developed bioplatform

The selectivity of the developed bioplatform was checked against non-targeted biological compounds, commonly detected in serum matrices but also belonging to the same family as the target ARA and with potential roles in inflammatory events.

**Fig. 4:**
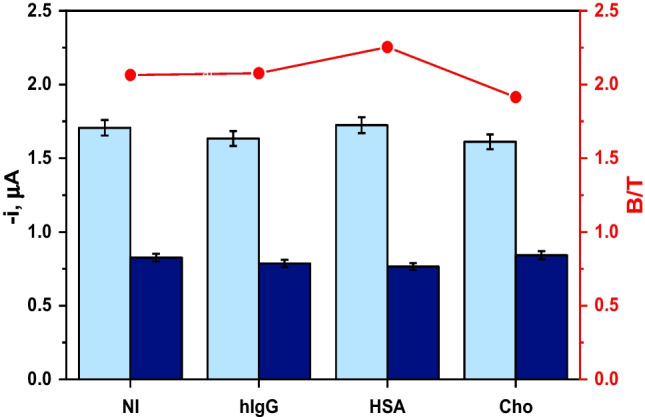
Amperometric measurements obtained with the developed bioplatform for 0.0 (B, light blue bars) and 5.0 µg mL^−1^ (T, dark blue bars) ARA standards prepared in the absence (NI) and in the presence of 1.0 mg mL^−1^ hIgG, 50 mg mL^−1^ HSA, and 2.5 mg mL^−1^ Cho, as well as the corresponding B/T ratios (red dots connected by lines)

With this purpose, the amperometric responses for 0.0 (blank, B) and 5.0 µg mL^−1^ (target, T) ARA standards were measured in the absence and in the presence of non-target biomolecules (human IgG, human serum albumin (HSA), and cholesterol (Cho)), at their normal concentration levels in serum. As Fig. 4 shows, none of the compounds affected the determination of ARA at the concentration levels tested, thus confirming the suitability of the developed bioplatform, in terms of selectivity, for the analysis of serum samples.

### Application to the analysis of serum samples from healthy individuals and SARS-CoV-2 and RSV-infected patients

To evaluate the potential of the developed bioplatform in the analysis of ARA in serum and to assist in the management of viral infections, selected cohorts of 10 healthy subjects, 10 patients diagnosed with SARS-CoV-2, and 10 diagnosed with RSV, were analyzed.

As shown in Table S1, a 1/10 sample dilution ensured the absence of matrix effect (Table S1). Moreover, due to the sensitivity of the method, the amperometric responses obtained for the diluted samples fell within the linear range provided by the bioplatform. Accordingly, the determination of ARA was performed by simple interpolation of the amperometric responses measured for the 10 times diluted samples into the calibration graph constructed with ARA standards (Fig. 3).

**Fig. 5:**
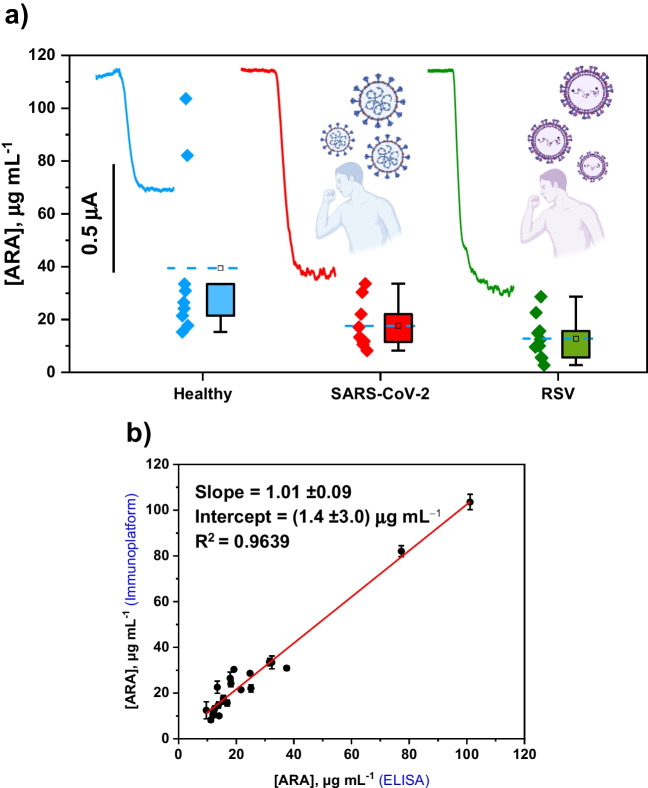
Boxplots displaying the ARA concentrations measured by the bioplatform in serum samples grouped into pools of healthy individuals and patients with SARS-CoV-2 and RSV, and representative amperograms of each type of sample (**a**). Correlation plot between the concentrations provided by the developed immunoplatform and the commercial ELISA kit (**b**)

The results obtained for 3 replicates (three different aliquots of the same sample analyzed with three different bioplatforms) are summarized in Table 3 and displayed in Fig. 5.

**Table 3:** ARA concentration in serum ($*mean value \pm ts/\sqrt{n}$; *n* = 3; α = 0.05) provided by the bioplatform for the different analyzed samples

Individual	Sample	[ARA], µg mL^−1*^	RSD_*n* = 3_, %
Healthy	1	31 ± 2	2.9
	2	82 ± 6	2.9
	3	27 ± 7	10.2
	4	24 ± 4	5.8
	5	ND	--
	6	33 ± 7	8.4
	7	21 ± 2	2.8
	8	104 ± 8	3.2
	9	15 ± 4	10.2
	10	18 ± 3	5.6
SARS-CoV-2	11	ND	--
	12	12 ± 2	5.7
	13	11 ± 3	12.4
	14	34 ± 4	4.8
	15	22 ± 4	7.2
	16	17 ± 5	11.0
	17	8 ± 2	11.0
	18	12 ± 4	15.5
	19	13 ± 3	7.9
	20	30 ± 1	1.4
RSV	21	6 ± 8	57.1
	22	16 ± 3	8.1
	23	10 ± 1	5.6
	24	13 ± 9	29.8
	25	29 ± 2	2.6
	26	5 ± 2	15.2
	27	10 ± 3	12.9
	28	15 ± 3	9.1
	29	3 ± 3	34.6
	30	23 ± 7	11.5

*ND, *non-detectable

The obtained results show significantly lower ARA concentration in serum of patients diagnosed with SARS-CoV-2 and RSV compared to healthy individuals. This agrees with numerous reports on the relationship between ARA metabolism and COVID-19 infection and lower serum ARA levels in infected patients [9–11, 13, 14, 17–20, 22]. Although lower ARA concentrations in serum have been reported also in SARS and MERS and other similar enveloped viruses [11, 16–20], these are the first results for ARA concentration levels in RSV-infected patients. It is important to remark that the ARA concentrations measured for healthy individuals agree well with that reported previously (mean level of 46.4 µg mL^−1^) [36], thus confirming the accuracy of the bioplatform for the analysis of serum samples. The correlation graph (Fig. 5b) between the results provided with the developed bioplatform and the commercial ELISA kit showed slope and intercept values that include 1 and 0, respectively, thus confirming the good agreement of the results provided by both methodologies.

The analysis of the results obtained using ROC curves and Mann-Whitney U test is shown in Fig. S4 (in the Supporting Information) and Table 4. The ROC curves confirmed the potential of ARA measurements to significantly discriminate between healthy and SARS-CoV-2 or RSV-infected individuals (Mann-Whitney U test < 0.033, AUC higher than 82.7%, and specificity and sensitivity higher than 77.8%). Conversely, ARA measurements cannot be used to discriminate between SARS-CoV-2 and RSV-infected individuals (Mann-Whitney U test 0.25, AUC of 60%. and sensitivity and specificity of 90 and 33%, respectively). According to these results, the following cut-off values for ARA concentration in serum to discriminate between healthy individuals and SARS-CoV-2 or RSV-infected individuals were 14 µg mL^−1^ and 17 µg mL^−1^, respectively.

It is important to note that many studies correlate the serum levels of ARA with the susceptibility and severity of certain individuals to respond to viral infections [8, 11, 16–20]. Therefore, the developed bioplatform may be applied, in addition to the management of viral infections, to the implementation of personalized vaccination strategies.

**Table 4:** Parameters of the ROC curves and the Mann-Whitney U test and cut-off values established in serum to detect SARS-CoV-2 and RSV infections according to the ARA concentration in serum

Parameter	Healthy vs. SARS-CoV-2	Healthy vs. RSV	SARS-CoV-2 vs. RSV
AUC (%)	82.7	94.4	60.0
Sensitivity (%)	77.8	100.0	90.0
Specificity (%)	77.8	77.8	33.3
Cut-off, µg mL^−1^	14.0	17.0	10.5
Mann-Whitney U test	0.033	0.004	0.25

## Conclusions

The first bioplatform involving a direct competitive immunoassay for the determination of the biomarker related to viral infection, ARA, in serum samples from individuals infected with SARS-CoV-2 or RSV is reported in this work. The designed method benefits from the combination of MµBs and SPCEs as efficient scaffold supports and signal transducers, respectively, ensuring the achievement of the analytical and operational characteristics required for its successful exploitation in real clinical scenarios. Based on the robust agreement between the results provided by the developed immunoplatform and those reported by employing completely different analytical methodologies, there is no room for doubt about the potential usefulness of the developed bioplatform for the sensitive, rapid, trustful, and affordable analysis of the target biomarker in clinical settings.

What is more important, apart from the globally accepted capabilities that MµBs- and SPEs-based electrochemical biosensing architectures can boast, including easy handling, portability, and point-of-care devices coupling, among others, the developed bioplatform allows its usage as a biosensing tool to manage ARA-related viral infectious processes, as well as to customize and evaluate the effectiveness of personalized vaccination strategies.

### Electronic supplementary material

Below is the link to the electronic supplementary material.

Supplementary Material 1

## Data Availability

The authors confirm that the data supporting the findings of this study are available within the article and its supplementary materials.
