# Supplementary material for: Contributing to the management of viral infections through simple immunosensing of the arachidonic acid serum level
Source: Mikrochim Acta. 2024 Jun 4;191(7):369. doi: 10.1007/s00604-024-06440-y (PMC11150294; doi:10.1007/s00604-024-06440-y)
Supplement: Supplementary file 1 — Supplementary Material 1 [file 604_2024_6440_MOESM1_ESM.docx]

**SUPPORTING INFORMATION**

**Contributing to the management of viral infections through simple immunosensing of the arachidonic acid serum level**

Rebeca M. Torrente-Rodríguez^1,†^, Víctor Ruiz-Valdepeñas Montiel^1, †^, Simona Iftimie^2^, Ana Montero-Calle^3^, José M. Pingarrón^1^, Antoni Castro^2^, Jordi Camps^4^, Rodrigo Barderas^3,5^, Susana Campuzano^1,*^, Jorge Joven^4^

*^1^Departamento de Química Analítica, Facultad de CC. Químicas, Universidad Complutense de Madrid, Pza. de las Ciencias 2, 28040-Madrid, Spain*

*^2^Servei de Medicina Interna, Hospital Universitari de Sant Joan, Institut d’Investigació Sanitària Pere Virgili, Universitat Rovira i Virgili, Av. Dr. Josep Laporte 2, 43204-Reus, Spain*

*^3^Chronic Disease Programme, UFIEC, Instituto de Salud Carlos III, 28220 Majadahonda, Madrid, Spain*

*^4^Unitat de Recerca Biomèdica, Hospital Universitari de Sant Joan, Institut d’Investigació Sanitària Pere Virgili, Universitat Rovira i Virgili, Av. Dr. Josep Laporte 2, 43204-Reus, Spain*

*^5^CIBER of Frailty and Healthy Aging (CIBERFES), Madrid, Spain*

*to whom correspondence should be addressed: [susanacr@quim.ucm.es](mailto:susanacr@quim.ucm.es)

^†^ These authors contributed equally to this work

| **CONTENTS** | **PAGE** |
| --- | --- |
| **Fig. S1** | **S2** |
| **Fig. S2** | **S3-S4** |
| **Fig. S3** | **S4** |
| **Fig. S4** | **S4** |
| **Table S1** | **S5** |
| **References** | **S5** |


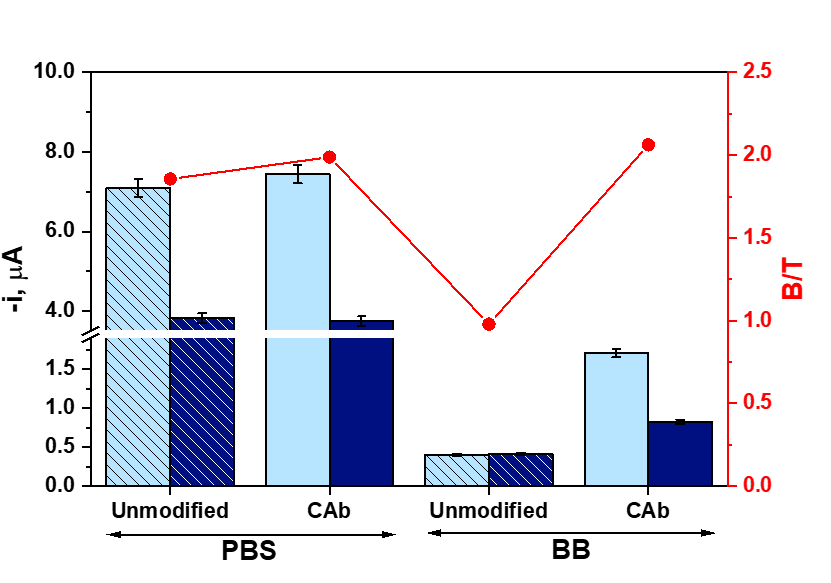


**Fig. S1** Influence of the buffer solution on the assay suitability. Variation of the amperometric responses obtained with the developed bioplatforms in PBS and commercial BB onto unmodified (patterned bars) or Ab-modified (non-patterned bars) for 0.0 (light blue) and 5.0 µg mL^−1^ (dark blue) of ARA standards, and the resultant B/T ratio (red dots connected by lines).

**Fig. S2** Amperometric responses obtained with the developed bioplatform for 0.0 (blank, B, white bars) and 5.0 µg mL^−1^ (target, T, grey bars) ARA standards and the corresponding B/T ratios (red dots connected by lines) by varying the volume of HOOC-MµBs (a), concentration (b) and incubation time of anti-ARA Ab (c), steps of the immunoassay (d), concentration of biotin-ARA competitor (e), incubation time of the competition step between free ARA and biotin-ARA competitor (f), and dilution (g) and incubation time (h) of the Strep-HRP enzymatic conjugate. The tentatively selected initial assay conditions were 3.0 µL HOOC-MµBs, 25 µg mL^−1^ anti-ARA Ab/45 min incubation time, 2.5 µg mL^−1^ biotin-ARA competitor/30 min incubation time, and 1/1000 Strep-HRP/30 min incubation time, and for each optimization study just the variable under study was ranged while the other were kept constant.

**Fig. S3.** Nyquist plots recorded for 5 mM [Fe(CN)_6_]^−3/−4^ in 0.1 M KCl solutions for a bare SPCE and SPCEs after trapping 1, 2, 3 and 5 μL of anti-ARA Ab-HOOC-MµBs. Range of frequencies: 10^5^–0.04 Hz; open circuit.

**Fig. S4** Potential of the ARA concentration in serum measured with the developed immunoplatform to detect the presence of SARS-CoV-2 or RSV infections. The analyzed results by ROC curves analyses are those summarized in Table 4.

**Table S1** Slope values of the resulting linear least-squared regression (in nA µg^−1^ mL) calculated from the calibration plots constructed from the amperometric responses obtained with the bioplatform for the determination of ARA standards prepared in buffered solution and in a 10-times diluted SARS-CoV-2 representative serum sample.

| Matrix | Slope | t_exp**_ | t_tab(0.05, 4, 2 tailed)**_ |
| --- | --- | --- | --- |
| Buffered solutions | (−185 ± 68) | -- | 4.303 |
| 10-times diluted serum of COVID-19 patient | (−171 ± 26) | 0.193 |  |

***Estimated as described in* [1].

**References**

1. Andrade JM, Estévez-Pérez MG (2014) Statistical comparison of the slopes of two regression lines: A tutorial. Anal Chim Acta 838:1–12. <https://doi.org/10.1016/j.aca.2014.04.057>.
